# Incidence and correlates of hepatitis C virus infection in a large cohort of prisoners who have injected drugs

**DOI:** 10.1186/1471-2458-14-830

**Published:** 2014-08-11

**Authors:** Kathryn J Snow, Jesse T Young, David B Preen, Nicholas G Lennox, Stuart A Kinner

**Affiliations:** Melbourne School of Population and Global Health, University of Melbourne, Melbourne, Australia; Centre for Health Services Research, School of Population Health, University of Western Australia, Perth, Australia; National Drug Research Institute, Curtin University, Perth, Australia; Queensland Centre for Intellectual and Developmental Disability, University of Queensland, Brisbane, Australia; School of Psychology, University of Queensland, Brisbane, Australia; School of Public Health and Preventive Medicine, Monash University, Melbourne, Australia; Centre for Adolescent Health, Murdoch Children’s Research Institute, Melbourne, Australia

**Keywords:** Hepatitis C virus, HCV, Prison, Injecting drug use, Incarceration

## Abstract

**Background:**

Hepatitis C virus (HCV) infection is common among prisoners, particularly those with a history of injecting drug use (IDU). Incarcerated people who inject drugs frequently report high-risk injecting practices both in prison and in the community. In spite of rising morbidity and mortality, utilisation of HCV-related services in Australia has been persistently low. This study aimed to describe the incidence, prevalence and correlates of HCV seropositivity in a large cohort of prisoners who have injected drugs, and to identify correlates of receiving confirmation of active infection.

**Methods:**

Data-linkage to a State-wide statutory notifiable diseases surveillance system was used to investigate the incidence of notified HCV seropositivity, seroconversion and confirmed HCV infection in a cohort of 735 prisoners with a history of IDU, over 14 years of follow up. Hepatitis C test results from prison medical records were used to identify correlates of testing positive in prison.

**Results:**

The crude incidence of HCV notification was 5.1 cases per 100 person-years. By the end of follow up, 55.1% of the cohort had been the subject of a HCV-related notification, and 47.4% of those tested in prison were HCV seropositive. In multivariable analyses, injecting in prison was strongly associated with HCV seropositivity, as was opioid use compared to injection of other drugs. The rate of reported diagnostic confirmation among those with notified infections was very low, at 6.6 confirmations per 100 seropositive participants per year.

**Conclusions:**

Injecting drugs in prison was strongly associated with HCV seropositivity, highlighting the need for increased provision of services to mitigate the risk of transmission within prisons. Once identified as seropositive through screening, people with a history of IDU and incarceration may not be promptly receiving diagnostic services, which are necessary if they are to access treatment. Improving access to HCV-related services will be of particular importance in the coming years, as HCV-related morbidity and mortality is increasing, and next generation therapies are becoming more widely available.

## Background

Hepatitis C virus (HCV) infection is a leading cause of cirrhosis, liver failure and liver cancer worldwide [1, 2]. The most common risk factor for HCV exposure in high-income countries is unsafe injecting drug use (IDU) [3, 4], and due to the close association between IDU and incarceration, HCV infection is highly prevalent among prisoners [5, 6].

Surveys of prisoners in several Australian states have recorded HCV seroprevalence in the range of 21–58% of all prisoners [7–11], and 42–77% of prisoners reporting a history of IDU [7, 8, 11]. Comparable prevalence estimates have been observed in prisoners who have injected drugs in Scotland, Spain, and the United States [6, 12, 13]. Seroconversion among HCV-naïve prisoners who inject drugs has been observed at rates between 5 and 34 cases per 100 person-years [8, 9, 14], with the highest rates recorded among those continuously incarcerated [5, 15].

Injecting drugs in prison presents an especially high risk of HCV transmission, due to the high prevalence of HCV infection and inadequate harm reduction services in many settings [5, 9]. There are as yet no needle and syringe exchange programs available in Australian prisons [16], and opioid substitution treatment is not available to men incarcerated in Queensland [17, 18], the state from which our participants were recruited. It is Queensland Corrective Services policy that all prisoners are offered testing for blood borne viruses including hepatitis C during health assessments at reception, however they may refuse testing [19].

Diagnosis of HCV infection is a two-step process. A positive result upon serological testing indicates a history of HCV exposure, and polymerase chain reaction (PCR) for viral nucleic acid confirms the presence of active (usually chronic) infection [20]. PCR confirmation may be foregone under some circumstances, however it forms a part of the standard diagnostic workup [20], and as such receipt of this test may be considered an indicator of engagement with care for HCV infection, a precondition for accessing treatment. It has recently been shown that a substantial proportion of seropositive individuals in New York do not receive PCR testing [21].

The uptake of treatment for HCV infection has been persistently low in Australia: despite the fact that treatment is fully subsidised, fewer than 1% of an estimated 230,000 Australian residents living with chronic HCV infection access treatment annually [3]. Access to HCV-related services is limited by a lack of knowledge about HCV infection in primary care [22], limited capacity and long waiting lists at some hospital-based services [23], and patient decisions to defer or forego potentially arduous therapy [24].

It has previously been shown that referral to HCV-related services among seropositive people who inject drugs (PWID) in Australia is particularly suboptimal [25]. As a substantial proportion of people living with HCV infection pass through prisons each year [26], identifying risk factors for non-engagement with diagnostic services in this population may assist efforts to improve access to treatment.

The present study aimed to a) describe the incidence of notified HCV seropositivity in a cohort of Australian prisoners with a history of IDU, including seroconversion among HCV-naïve participants after release from prison, b) identify correlates of testing positive for HCV in prison, and c) identify correlates of receiving notified PCR confirmation of active infection.

## Methods

### Cohort and study design

The Passports cohort consists of 1,325 individuals recruited to a randomised controlled trial of a service brokerage intervention prior to release from seven prisons in Queensland, Australia. Potentially eligible participants were identified from prison records and included sentenced prisoners within six weeks of expected release from custody (full-time or parole) who were able to provide informed, written consent. Women were oversampled to ensure sufficient numbers for stratified analyses. According to data provided by Queensland Corrective Services, the cohort is otherwise representative of the population of people released from prisons in Queensland over the same period [27].

Baseline data were collected via face-to-face administration of a structured questionnaire in confidential interviews between August 2008 and July 2010. The data collection tool covered demographic characteristics; incarceration history; general health, mental health and health-related quality of life; alcohol, tobacco and other drug use prior to and during incarceration, including history of IDU; and other health risk behaviours. A detailed description of the recruitment and interview process has been published previously [27].

### Measures and data sources

Variables of interest in this study were sex; age (categorised by birth cohort); level of education (<10 vs. ≥10 years); Indigenous status (yes/no); time since initiation of injecting; lifetime injection of heroin (vs. injection of other drugs) and history of opioid substation therapy (OST); history of tattooing (vs. no tattoos); prior incarceration (juvenile and/or adult vs. none); lifetime prison IDU (vs. community only); and lifetime sharing of injecting equipment such as needles, syringes, spoons or tourniquets (vs. no reported sharing).

In our cohort, injecting in prison was reported almost exclusively by participants who also reported sharing equipment in the community [8], making them a subset of this larger group. To accommodate this, these two variables were combined into one ordinal variable with 3 categories (1: no prison IDU and no sharing in the community; 2: sharing in the community without prison IDU; 3: sharing in the community and prison IDU).

Outcomes of interest were HCV seropositivity and PCR-confirmed active HCV infection. Clinical identification of both HCV seropositivity and active HCV infection are notifiable events in Australia, with reports made to state health authorities. Each notification event appears in the dataset as a separate record, including a personal unique identifier, sex, diagnosis, type of test (serology or PCR) and test date. Surveillance data were obtained via retrospective, probabilistic record linkage to the Queensland Notifiable Conditions Systems for the period from 1^st^ January 1999 to 1^st^ June 2013. Linkage was based on full name (including all known aliases), sex, date of birth, and postcodes of residence. The linkage procedure was based on that used by the Western Australian Data Linkage System, and has been validated previously [28]. To permit censoring, data on deaths between recruitment and 31 May 2013 were obtained via probabilistic linkage to the Australian National Death Index.

Of the original Passports cohort, seven (0.5%) participants withheld consent for record linkage, and three (0.2%) declined to answer questions regarding IDU at baseline interview. Their data were not included in this study. The final sample described here consists of 735 participants who reported a history of IDU, representing 55.5% of the entire Passports cohort. Among this sample, 419 participants had a record of a test for HCV antibodies in prison. Results were available for 403 participants, and these were extracted from their prison medical records after their baseline interview.

### Statistical methods

The incidence of HCV notification was calculated among all those who had injected drugs. Participants estimated to have initiated IDU after 1999 were considered at risk from an estimated date of first IDU, based on self-reported age at initiation. The rate of apparent seroconversion after release from prison was calculated in a subset of individuals whose prison medical records included a negative test for HCV in the year prior to their release. These participants were deemed to be at risk from the date of their release from prison, and were censored on the date of their first notification for HCV, their date of death, or 1 June 2013, whichever came first.

Logistic regression was used to estimate the association between measured risk factors and a positive HCV test in prison. Year of birth was highly correlated with time since initiation of injecting; consequently, only time since initiation was included in the model, as this is the more direct measure of time at risk of HCV exposure. Interactions between age, sex, time since initiation and opioid use were explored and their impact on the multivariate model assessed. No interactions were found to be significant. Of the 403 participants with test results available, 4 did not answer all interview questions, resulting in a final sample of 399 for this analysis.

In order to investigate the delay between positive serological testing and diagnostic (PCR) testing, the rate of notified PCR confirmation of active infection was calculated among those with a record of notified HCV seropositivity. As the outcome of interest was the delay between detection of HCV antibodies and PCR confirmation of active infection, 28 participants whose first notification was based on PCR rather than serological testing were excluded from PCR confirmation analysis, providing a final sample of 377 participants for this analysis. Follow-up time for each participant began on the date of first notification for HCV seropositivity. Censoring occurred at the test date for the first notified PCR confirmation, date of death, or 1 June 2013, whichever came first.

Potential correlates of PCR confirmation were investigated using Cox regression. Due to the smaller number of observations available for this analysis, continuous variables were divided above and below the median value to preserve statistical power, and the two higher-risk injecting categories were combined into a single category (sharing equipment and/or injecting in prison, vs. no sharing equipment and no prison IDU).

Exposures and potential confounders of interest for both analyses were identified during a review of the literature. Possible causal relationships between these variables were assessed using directed acyclic graphs, and this informed the specification of the multivariate models. Both models were specified in advance and included all variables identified as exposures and/or potential confounders. Age and time since initiation were highly correlated, and were not included in the same models: time since initiation was included in the analysis of prison test results, as it is the more direct measure of time at risk of HCV exposure, while age was used in the PCR analysis as it is of higher clinical relevance among people living with HCV infection. All statistical analyses were performed in Stata 13 (StataCorp LP, Texas, USA).

### Ethics approval

Ethics approval for the study was granted by The University of Queensland’s Behavioural and Social Sciences Ethical Review Committee. Approval for linkage to data from the National Death Index and the Queensland Notifiable Conditions Systems were provided by the Australian Institute of Health and Welfare Ethics Committee and the Queensland Health Human Research Ethics Committee respectively.

## Results

The injecting cohort was predominantly young (median age 29 years) and male (77.0%), with a median time since initiation of IDU of 16 years. A large majority (86.7%) had been incarcerated previously, and having injected drugs in prison was reported by 285 participants (39.2%). The majority of the sample (94.8%) reported having been tested for HCV, and 405 (55.1%) had a record of a HCV-related notification. Prison medical records indicated that 419 participants (60.2%) had been tested in prison; of the 403 participants for whom results were available, 191 (47.4%) were seropositive.

The crude incidence of HCV notification in the cohort between 1999 and mid 2013 was 5.1 per 100 person-years (see Table 1). The incidence of seroconversion among the 77 individuals who were confirmed as HCV seronegative in the year prior to release from prison was 5.5 per 100 person-years; the median time to first HCV notification for the 12 individuals who seroconverted was 1 year, 11 months.

**Table 1:** **Incidence of notified HCV seropositivity and PCR-confirmation**

Group	Participants at risk	Person time (years)	Events	Incidence rate per 100 person years (95% confidence Interval)
Injecting cohort	735	7,919	405	5.1 (4.6, 5.6)
Seroconversion after release*	77	266	12	5.5 (3.1, 9.7)
PCR confirmation after notification	377	1,968	130	6.6 (5.6, 7.8)

*Among participants who received a negative test result in prison in the year prior to release.

In the multivariable analysis there was a strong association between heroin injection and testing positive for HCV in prison, particularly if a history of OST was also reported (see Table 2). Sharing injecting equipment in the community and injecting in prison were associated with increased risk, as was greater time since initiation of injecting. Women and people who had been incarcerated as juveniles were substantially more likely to test positive.

**Table 2:** **Correlates of HCV seropositivity in injecting cohort with in prison test results**

	N participants (% of those answering interview question)	N seropositive (% of category)	Univariate odds ratio (95% confidence interval)	Multivariable odds ratio (95% confidence interval)
Female (vs. male)	191 (21.8)	54 (61.4)	2.06 (1.27, 3.35)	2.74 (1.45, 5.18)
Indigenous (vs. non-indigenous)	91 (22.6)	40 (44.0)	0.84 (0.53, 1.35)	0.76 (0.42, 1.36)
Educated 10 years or more (vs. <10 years)	196 (48.6)	86 (43.4)	0.73 (0.49, 1.08)	0.79 (0.48, 1.29)
Injecting 10 years or more (vs. <10 years)	210 (52.2)	97 (46.2)	3.03 (1.66, 5.54)	2.83 (1.38, 5.78)
Injecting 20 years or more (vs. <10 years)	115 (28.6)	76 (66.1)	6.88 (3.55, 13.34)	4.54 (2.05, 10.1)
Heroin injection (vs. other drugs)	163 (40.8)	87 (53.4)	3.66 (2.25, 5.94)	1.95 (1.11, 3.43)
Heroin injection with OST (vs. other drugs)	86 (21.5)	66 (76.7)	10.5 (5.64, 19.7)	4.13 (2.02, 8.42)
Shared injecting equipment in community (vs. no sharing, no prison IDU)	109 (27.1)	64 (58.7)	4.91 (2.83, 8.53)	3.19 (1.69, 6.04)
Injected in prison (vs. no sharing, no prison IDU)	156 (38.7)	96 (61.5)	5.52 (3.30, 9.23)	3.57 (1.94, 6.57)
Tattoos (vs. no tattoos)	340 (84.4)	171 (50.3)	2.18 (1.23, 3.85)	1.38 (0.70, 4.98)
Prior incarceration as adult only (vs. none)	209 (51.9)	99 (47.4)	2.70 (1.30, 5.63)	2.05 (0.84, 4.98)
Prior incarceration as juvenile (vs. none)	150 (37.2)	81 (54.0)	3.52 (1.66, 7.49)	3.07 (1.20, 7.88)

Of the 377 participants with an initial HCV notification based on serology, only 130 (34.5%) had a record in the notification data for PCR confirmation of active infection (see Table 3). The rate of PCR confirmation after first HCV notification was low, at 6.6 confirmations per 100 seropositive participants per year (95% CI: 5.6, 7.8). For the 130 participants with PCR confirmations, the median time between first notification and first confirmation was 1 year, 11 months. Female sex was associated with a higher rate of confirmation in univariable analysis, however this effect was attenuated in the multivariable analysis. Only juvenile incarceration remained significantly associated with reduced PCR confirmation in the multivariable model.

**Table 3:** **Correlates of PCR confirmation in HCV seropositive participants**

	Person years	PCR confirmations	Rate (per 100 person years)	Univariate hazard ratio (95% CI)	Multivariate hazard ratio (95% CI)
Total	1,968	130	6.6	-	
Female (vs. male)	418	41	9.8	1.65 (1.14, 2.39)	1.36 (0.91, 2.03)
Born <1979 (vs. 1979 or later)	984	55	5.6	0.81 (0.57, 1.15)	0.84 (0.59, 1.21)
Educated 10 years or more (vs. <10 years)	910	59	6.5	0.96 (0.68, 1.36)	0.88 (0.61, 1.27)
Indigenous (vs. non-indigenous)	440	31	7.1	1.08 (0.72, 1.61)	1.11 (0.72, 1.70)
Ever injected heroin (vs. only injected other drugs)	1,499	86 (33.6)	5.7	0.66 (0.45, 0.94)	0.68 (0.46, 1.00)
Higher risk injecting (vs. no sharing or prison IDU)	1,510	100 (35.8)	6.6	1.05 (0.70, 1.58)	1.21 (0.79, 1.86)
Previously incarcerated –adult only (vs. never)	1,018	73 (37.8)	7.2	0.59 (0.35, 1.00)	0.64 (0.37, 1.10)
Previously incarcerated –juvenile (vs. never)	817	39 (26.5)	4.8	0.40 (0.23, 0.70)	0.44 (0.24, 0.80)

## Discussion

### Incidence, prevalence and correlates of HCV exposure

At 47.4%, the prevalence of HCV seropositivity among participants tested in prison is within the range previously reported among incarcerated Australians who have injected drugs [7, 11, 26]. The crude incidence rate reported here is not directly comparable to other cohorts, as the retrospective component of this study meant that the majority of follow-up time preceded incarceration. The rate of seroconversion in the community after release among those with a history of IDU was similar to that reported in two comparable Australian cohorts of ex-prisoners [14, 29], and in a cohort of PWID continuously incarcerated in a Spanish prison with a needle and syringe exchange program [13]. However, it was substantially lower than rates recorded among continuously incarcerated PWID in New South Wales, where needle and syringe exchange programs are not available [5, 15]. As in other studies, female participants were significantly more likely to test positive for HCV in prison, and the observed HCV seroprevalence of 61.3% among female participants tested in prison is within the range previously reported among incarcerated Australian women who inject drugs [7–9, 26].

The high prevalence of methamphetamine injection relative to heroin injection in Queensland has been reported previously, as has a higher prevalence of HCV exposure among recent heroin injectors relative to recent methamphetamine injectors [26]. The increase in risk of HCV seropositivity among heroin injectors compared to injectors of other drugs in our cohort was substantial. Heroin injection was strongly associated with a number of additional risk factors for HCV exposure including prison IDU, sharing injecting equipment in the community, and younger age at first IDU (data not shown). That the increased risk of HCV exposure was preserved after adjustment for these factors may reflect variation in unmeasured behaviours such as frequency of injecting, frequency of sharing injecting equipment, and the number of people with whom injecting equipment was shared.

This variation in unmeasured aspects of risky injecting may also explain the observed association between a history of OST and higher odds of HCV seropositivity, which has been reported previously [5]; a history of OST may indicate a higher level of heroin dependence, and consequently may be associated with a history of more frequent or more prolonged risky injecting. In our sample, 86.2% of those with a history of OST reported higher risk injecting practices (sharing equipment and/or injecting in prison), compared to 74.9% of heroin users with no history of OST, and 38.7% of injectors of other drugs.

As in other studies [7, 14, 15], prison IDU stands out as a key correlate of HCV exposure. Injecting in prisons might be reduced if OST were available to all opioid-dependent inmates; women in our cohort, who may have access to OST while incarcerated in Queensland, were far less likely to report having injected in prison than men were (16.6% vs. 45.9%). The risk to those who do inject in prison might be reduced if additional harm reduction measures, such as needle and syringe exchange programs, were available.

### Incidence and correlates of PCR confirmation

Only one third (34.5%) of those with notified HCV seropositivity subsequently appeared in the notification data as having received PCR confirmation of active infection, suggesting that at least half of those participants living with chronic HCV infection may not have received complete diagnostic assessments. If all those with notified HCV exposure received PCR testing to confirm their current infection status within one year, assuming that 70-80% of those with notified HCV exposure went on to develop chronic infection [6], the expected rate of PCR confirmation would be no lower than 70 per 100 person years. The rate of confirmation observed in this cohort is an order of magnitude lower, at 6.6 confirmations per 100 seropositive participants per year.

The fact that older age was not associated with higher rates of PCR confirmation is concerning, as this may indicate that the presence of easily identifiable risk factors for greater duration of chronic HCV infection is not resulting in more attentive diagnostic follow-up in this population. However, the statistical power available for this analysis would have been limited by the low rate of confirmation, which is sobering in itself.

### Limitations

The passive nature of the surveillance system from which our data on incidence were drawn may have led to underestimation of the true incidence of HCV infection and seroconversion. Most previous studies in this population have used active serosurveys, while the present study used passively collected surveillance data to estimate incidence. As a result, the incidence estimates may have been influenced by the frequency of testing and the completeness of reporting. Any lengthy delays between seroconversion, testing and reporting would lead to an underestimate of the true incidence of HCV infection in the cohort.

Likewise, if positive PCR results are reported less consistently than positive antibody results, the incidence of PCR confirmation will have been underestimated. The strength of this data-linkage approach, however, is that any participant might appear in the surveillance data. Outcome ascertainment was not dependent on continued contact with investigators or return to prison, which can result in substantial and biased attrition [30].

Responses to questions regarding high-risk behaviour may have been affected by recall bias; participants who had tested positive for HCV in prison may have been more likely than those who knew that they were seronegative to report stigmatised, higher-risk injecting practices. This is unfortunately an unavoidable limitation of studies concerning stigmatised, illegal behaviour; ultimately, self-report is often the only way to collect such information [31]. That half of even our seronegative participants reported at least one higher risk injecting practice, however, suggests that this effect may not be very strong.

### Strengths

To the best of our knowledge, this study documents the incidence of HCV seropositivity in the largest cohort of prisoners with a history of IDU yet described in the global literature [32]. It is one of a small number of true cohorts recruited in prison settings, as opposed to repeated cross-sectional surveys of convenience samples, in which HCV incidence has been documented. As far as we could ascertain, this is the first cohort of people with a history of incarceration in which the incidence of PCR confirmation in the community has been documented.

## Conclusions

HCV exposure is highly prevalent among incarcerated PWID, and prisoners in Australia do not have access to the full range of harm reduction measures which are available to people who inject in the community [5, 15]. There is on-going discussion of trialling a needle and syringe exchange program in one prison in Australia [16], however this has not yet been implemented. That only 60% of participants who self-reported a history of IDU had a test for HCV in their prison medical records may indicate that increased efforts to provide testing are also needed, provided that the right of prisoners to refuse testing is respected. In addition, the current gaps in the provision of OST to opioid dependent people incarcerated in Australia should be addressed.

The low uptake of HCV treatment in Australia has long been recognised as a public health issue [3, 25, 33], and is becoming ever more pressing as the incidence of primary liver cancer rises [34–36]. Our findings indicate that clinical follow-up after detection of HCV antibodies in people with a history of incarceration may be very poor. A substantial proportion of people living with chronic HCV infection pass through prisons each year [37], and this provides an opportunity for intervention with a highly marginalised group who may not otherwise access HCV-related services [38].

Studies in other Australian settings have demonstrated that providing HCV treatment in prisons is effective [38]. With next generation therapies now becoming available, reduced treatment duration may make the provision of therapy to prisoners feasible even for those serving short sentences. In light of rising HCV-related morbidity and mortality in the community, increased efforts focusing on both prevention and treatment are needed to protect incarcerated PWID from HCV infection and its potentially fatal sequelae.
